# Adipocyte‐Derived Leptolin Enhances Energy Expenditure and Prevents Obesity

**DOI:** 10.1002/advs.202516081

**Published:** 2026-05-25

**Authors:** Jiarui Liu, Bingwei Wang, Zhijie Su, Xiaoxv Han, Miao He, Yun Zhao, Yujia Hou, Daotong Li, Weiguang Zhang, Lihua Qin, Ke Wang, Yanchun Li, Yi Yan, Siwang Yu, Xiaoshuai Huang, Tairan Yuwen, Jianyuan Luo, Ruimao Zheng

**Affiliations:** ^1^ Department of Anatomy Histology and Embryology School of Basic Medical Sciences Peking University Beijing China; ^2^ Department of Pharmaceutical Analysis and State Key Laboratory of Natural and Biomimetic Drugs School of Pharmaceutical Sciences Peking University Beijing China; ^3^ Biomedical Engineering Department Peking University Beijing China; ^4^ China Institute of Sport and Health Science Beijing Sport University Beijing China; ^5^ Sport Science College Beijing Sport University Beijing China; ^6^ State Key Laboratory of Natural and Biomimetic Drugs Department of Molecular and Cellular Pharmacology Peking University Beijing China; ^7^ Department of Medical Genetics Center for Medical Genetics Peking University Health Science Center Beijing China; ^8^ Neuroscience Research Institute Peking University Beijing China; ^9^ Key Laboratory for Neuroscience Ministry of Education/National Health Commission Peking University Beijing China; ^10^ Beijing Life Science Academy Beijing China

**Keywords:** adipokine, energy expenditure, exercise, fat mobilization, leptolin, mini‐leptolin treatment, obesity, *Tmem52*, white adipose tissue

## Abstract

Adipokines are key factors in regulating energy homeostasis. We identified a novel adipokine, which we named leptolin. In humans, leptolin levels in white adipose tissue were positively correlated with exercise and negatively associated with body mass index. Leptolin levels were positively correlated with lipolysis‐promoting gene expression. We observed elevated leptolin in serum from athletes and lower leptolin in serum from obese individuals. Leptolin gene‐knockout mice exhibited increased adiposity and body weight, and decreased energy expenditure. Transgenic leptolin overexpressing in mice showed obesity‐resistant phenotypes. Treatment with leptolin promoted fat mobilization, increased energy expenditure, and reduced body weight, without affecting food‐intake or motor activity. Leptolin is a novel adipokine with a capacity to improve metabolic status and may serve as a new therapeutic agent for obesity and metabolic disorders.

## Graphical abstract

We identified a novel adipokine, which we named leptolin. In humans, leptolin levels in white adipose tissue were positively correlated with exercise and negatively associated with body mass index. We observed elevated leptolin in serum from athletes and lower leptolin in serum from obese individuals. Leptolin may serve as a new therapeutic agent for obesity and metabolic disorders.

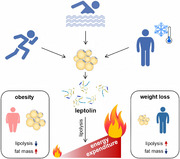

## 1 Introduction

Obesity, a chronic disease defined by excessive fat accumulation, has become an important cause of morbidity and mortality in humans [1]. Obesity results from the dysregulation of energy balance [2, 3]. Adipokines, proteins secreted by adipose tissue, play critical roles in the regulation of energy balance [4]. For instance, leptin activates hypothalamus to promote fat utilization, elevate energy expenditure (EE), reduce appetite and body weight [5, 6, 7]. Adiponectin stimulates fat mobilization and reduces fat accumulation [8, 9]. Irisin, an exercise‐induced adipokine/myokine, promotes lipolysis, induces white adipose browning, and enhances EE [10, 11]. Adipokines have potential for the treatment of obesity and related metabolic disorders [12]. Leptin replacement therapy improves body weight, endocrine function, and behavior in obese patients with leptin deficiency [13]. Adiponectin has anti‐obesity and insulin‐sensitizing effects [14]. Taken together, these lines of evidence reveal that adipokines may stimulate lipolysis, elevate EE, and improve metabolic status.

Physical exercise and cold exposure promote fat‐burning [15], induce the synthesis and secretion of anti‐obesity adipokines, such as adiponectin and irisin [10, 11, 16, 17]. Spiegelman and colleagues identified prosaposin as a novel adipokine by examining proteome changes with exercise and cold adaptation [18]. Villarroya and colleagues identified CXCL14 as a brown fat‐secreted adipokine through analysis of transcriptomic data from brown adipose tissue (BAT) in mice exposed to cold [19]. These “exerkines” have potential roles in improving metabolic health and treating obesity [20, 21]. In the present study, by analyzing transcribed genes in white adipose tissue (WAT) induced by running, swimming and cold exposure, we identified a novel anti‐obesity adipokine, the leptolin, encoded by the *Tmem52* gene. Leptolin enhances EE and prevents high‐fat diet (HFD)‐induced obesity, without affecting appetite and food intake. Leptolin may have the potential to treat obesity and related metabolic diseases.

## 2 Results

### 2.1 *Tmem52* is Induced by Exercise and Cold Exposure

**FIGURE 1:**
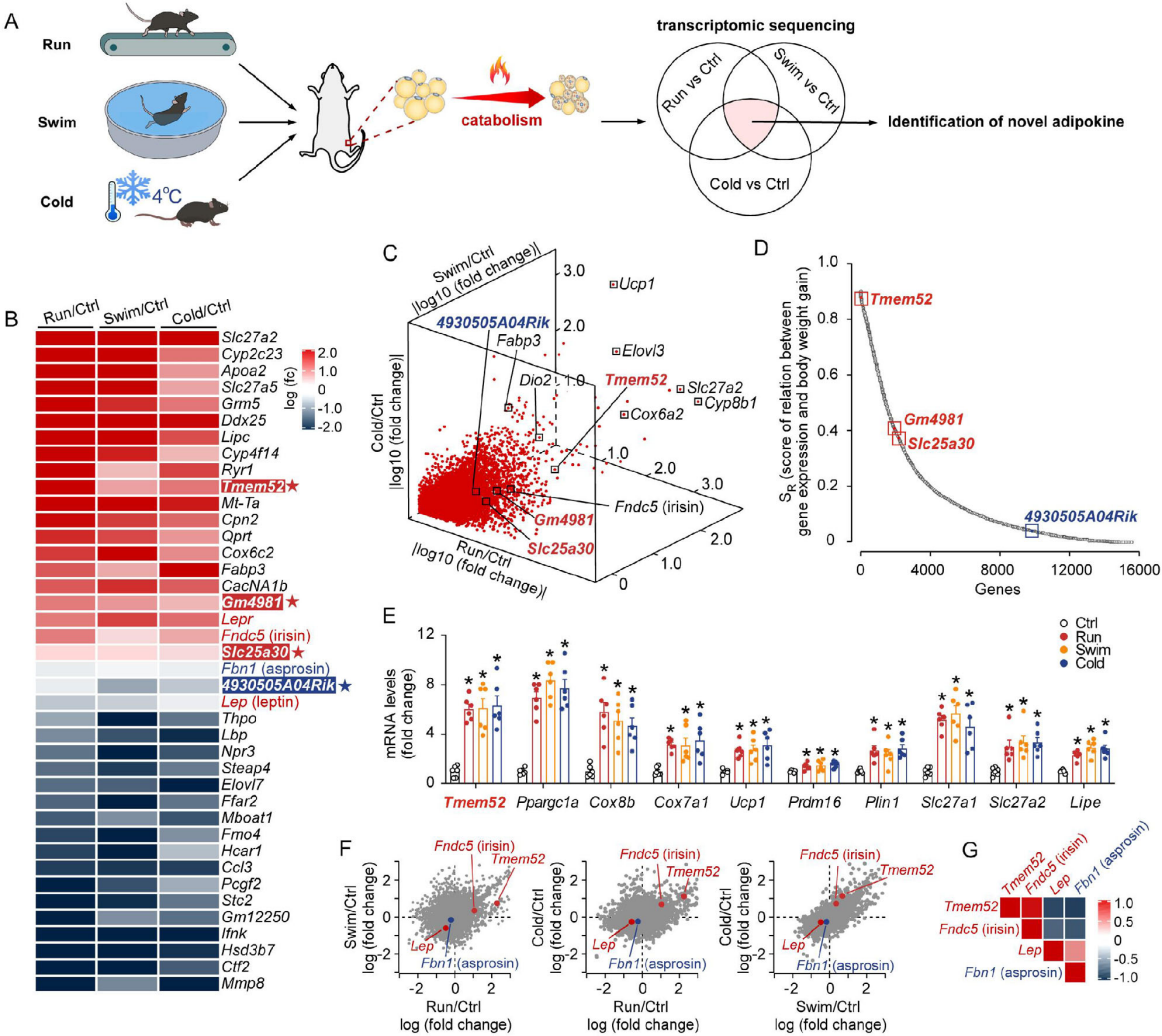
*Tmem52* is induced by exercise and cold exposure. (A) Schematic representation of the experimental workflow; RNAseq: n = 3 per group. (B) Heatmaps representing the selected 20 upregulated (red) and 20 downregulated (blue) genes—run versus ctrl group mice (left) swim versus ctrl group mice (middle) and cold versus ctrl group mice (right). (C) Three‐dimensional plot highlighting the differential expression genes (DEGs); each data point represents a gene. (D) Distribution of the calculated score (correlation between gene expression and body weight) of individual genes; the red dot represents *Tmem52*, *Gm4981*, and *Slc25a30* gene, the blue dot represents *4930505A04Rik* gene. (E) Relative mRNA expression of the indicated genes in iWAT; data are mean ± s.e.m.; *n*  =  6 per group; the one‐way ANOVAs were used for statistical analysis followed by Bonferroni's post hoc test; ^*^ *p* < 0.05. (F) Scatter plot highlights the DEGs of run, swim and cold compared with ctrl, significantly altered genes are colored in red or blue. (G) Heatmap showing correlation between mRNA levels of *Tmem52*, irisin, leptin, and asprosin. See also Figures S1 and S2.

We took advantage of observations that exercise and cold exposure induce secretion of bioactive proteins from adipose tissue (Figure 1A) [18, 19]. Genome‐wide transcriptome sequencing analysis showed that expression of genes associated with lipolysis (*Slc27a2, Slc27a3, Pdk4 and Plin5*) and mitochondrial function (*Ppargc1a, Cox7a1, Cox8b and Ucp1*) were upregulated; and expression of genes involved in lipid synthesis (*Cebpa and Srebf1*) and energy metabolism negative regulation (*Acvr2b, Npr3, Nr1d1* and *Tshr*) were downregulated in the inguinal white adipose tissue (iWAT) under these conditions (Figure S1A). Venn analysis showed that 424 genes were upregulated and 295 genes were downregulated (Figure S1B). Gene ontology (GO) and Kyoto encyclopedia of genes and genomes (KEGG) analyses showed that the pathways associated with lipolysis, β‐oxidation and mitochondrial function were activated (Figure S1C). Gene set enrichment analysis (GSEA) (Figure S1D,E) demonstrated that exercise and cold exposure promoted the lipid and glucose metabolism processes.

We observed that *Tmem52*, *Slc25a30*, *Gm4981*, and *4930505A04Rik*, four genes of unknown function, were among the top 20 upregulated or downregulated genes in response to exercise and cold exposure (Figure 1B,C). Notably, genetic correlation analysis [22] between obesity and over 16000 genes showed that *Tmem52* was one of the top‐ranked genes (Figure 1D). Real‐time quantitative PCR analysis validated that *Tmem52* and the mRNAs associated with mitochondrial function (*Ppargc1a, Cox8b, Cox7a1, Ucp1, Prdm16*) and fat mobilization (*Plin1, Slc27a1, Slc27a2*) were upregulated in response to exercise and cold exposure (Figure 1E). Messenger RNA for leptin (*Lep*) and asprosin (derived from the *Fbn1* gene) were decreased while irisin (derived from the *Fndc5* gene) was increased in iWAT under these fat‐burning conditions (Figure S1A and Figure 1F). Leptin, asprosin and irisin have previously been reported as being secreted from white adipose tissue under the condition of cold exposure and exercise [10, 11, 23, 24, 25, 26]. *Tmem52* gene expression was positively associated with irisin and negatively associated with leptin and asprosin (Figure 1G). Together, these findings suggest that *Tmem52* encodes an adipokine that could facilitate fat utilization and enhance energy expenditure.

### 2.2 *Tmem52* is an Obesity‐Related Gene, With Conserved Amino Acid Sequence in Mammals

To explore whether *Tmem52*, *Slc25a30*, *Gm4981*, and *4930505A04Rik* may be associated with adipose homeostasis, we created *Tmem52* gene‐knockout mice (*Tmem52*‐KO), *Slc25a30* gene‐knockout mice (*Slc25a30*‐KO), *Gm4981* gene‐knockout mice (*Gm4981*‐KO), and *4930505A04Rik* gene‐knockout mice (*4930505A04Rik*‐KO) (Figure S2A,C,E,G). Notably, we found that the body weight of *Tmem52*‐KO mice was remarkably higher than that of controls on a high‐fat diet (Figure S2B). In contrast, the body weight of *Slc25a30*‐KO, *Gm4981*‐KO, or *4930505A04Rik*‐KO mice was not markedly altered, as compared with controls (Figure S2D,F,H). These results revealed that *Tmem52* may serve as an anti‐obesity gene.

**FIGURE 2:**
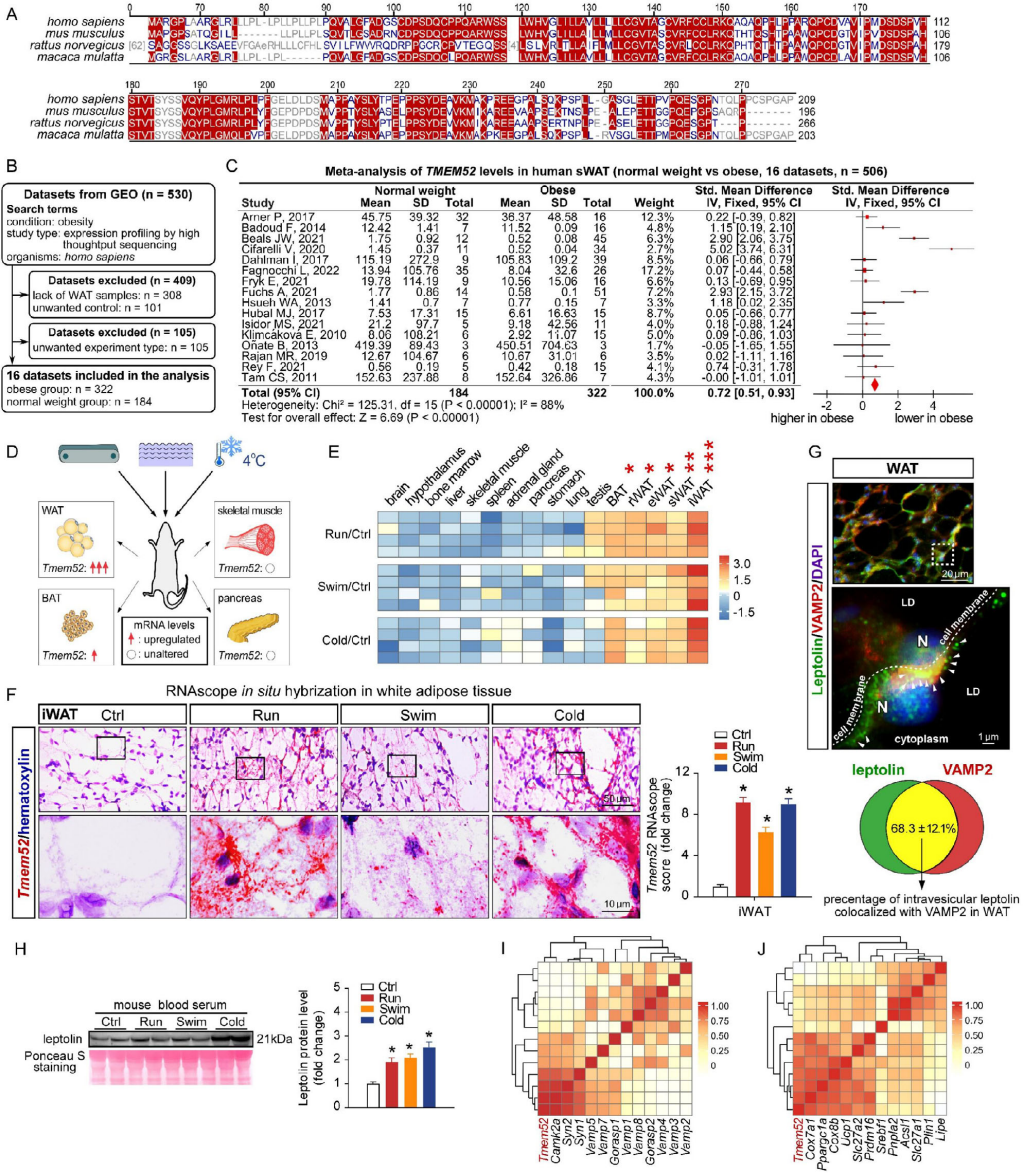
*Tmem52*‐encoded protein (leptolin) is identified as an adipokine, found in adipose tissue and blood serum. (A) Multiple alignment of leptolin of the 4 species (homo sapiens, mus musculus, rattus norvegicus, macaca mulatta). (B) Schematic illustration of screening workflow of normal weight/obese human sWAT datasets, 16 datasets are included in the meta‐analysis. (C) Forest plot of *TMEM52* mRNA levels in sWAT of normal weight/obese human datasets (normal weight, *n* = 184, obese, *n* = 322). (D) Schematic of *Tmem52* mRNA levels measurement in multiple organs and tissues of mice. (E) Relative mRNA expression (fold change) of *Tmem52* in multiple organs and tissues of mice in response to exercise and cold exposure; *n*  =  4 per group. (F) Representative RNAscope images visualizing *Tmem52* mRNA in iWAT; red dots were indicative of *Tmem52* mRNA signal; hematoxylin was used to stain the nucleus; scale bar, 50 µm (upper panel) and 10 µm (down panel). (G) Representative immunofluorescence images of leptolin and VAMP2 in iWAT; scale bar, 20 µm (upper panel) and 1 µm (down panel); and percentage of intra‐vesicular leptolin colocalized with VAMP2 in iWAT. (H) Representative immunoblots of leptolin and images of Ponceau S staining from mice serum; *n*  =  8 per group. (I) Heatmap showing correlation between *Tmem52* mRNA levels in iWAT and the genes related to the secretory function of adipocyte. (J) Heatmap showing correlation between *Tmem52* mRNA levels and genes associated with mitochondrial function and lipolysis. Data are mean ± s.e.m.; one‐way ANOVAs followed by Bonferroni's post hoc test were used for statistical analysis of (F) and (H); ^*^ *p* < 0.05. See also Figures S3–S8.

Phylogenetic analysis of the murine *Tmem52* gene [27] revealed evolutionarily conserved orthologs across all sequenced mammalian genomes, demonstrating remarkable amino acid sequence conservation (Figure S3A,B). Human and mouse *Tmem52*‐encoded protein (named as leptolin) are 77% identical (Figure 2A), compared to 100% identity for irisin, 83% identity for leptin, 83% identity for adiponectin and 59% identity for resistin, suggesting a conserved function of leptolin in human and mouse [10]. Computational signal peptide prediction analysis revealed strong evidence that the N‐terminal domain of leptolin contains a canonical secretory signal sequence.

These findings demonstrate that leptolin exhibits remarkable cross‐species sequence conservation, showing its potential physiological functions. Leptolin possesses a canonical N‐terminal signal peptide domain, and its molecular weight falls within the range of secreted proteins. Taken together, these observations imply that leptolin may be a novel protein involved in the regulation of metabolic function.

### 2.3 Leptolin is Identified as an Adipokine, Found in Adipose Tissue and Blood Serum

Meta‐analysis of the 16 obesity‐related human studies showed that *TMEM52* mRNA levels in subcutaneous WAT (sWAT) of obese individuals were lowered, as compared with that of normal weight individuals (*P* <  0.00001) (Figure 2B,C). In mice, *Tmem52* mRNA levels in fat pads, especially in iWAT, were elevated (6‐8‐fold) under the condition of exercise and cold exposure (Figure 2D,E and Figure S4A–C). Notably, the single‐cell level methodology of the HPA‐database provides strong evidence that *Tmem52* gene is top‐ranked and highly specific for subcutaneous adipocytes “TOP2” and visceral adipocytes “TOP3”, among all cell types in the human body (Figure S4D–F). In situ hybridization (RNAscope) revealed that *Tmem52* mRNA in iWAT was robustly enhanced (6‐9‐fold) (Figure S5A and Figure 2F). Moreover, using the global GEO datasets, we also found that exercise and cold exposure could elevate*Tmem52* expression in white adipose tissue (Figure S6A–D). Acute mechanical stress (cyclic mechanical stretch, 0.5 Hz, 10–21% elongation) could induce a remarkable alteration of the *Tmem52* mRNA expression level (Figure S6E). Whereas, fasting did not cause a remarkable alteration of *Tmem52* level in WAT (Figure S6F,G).

**FIGURE 3:**
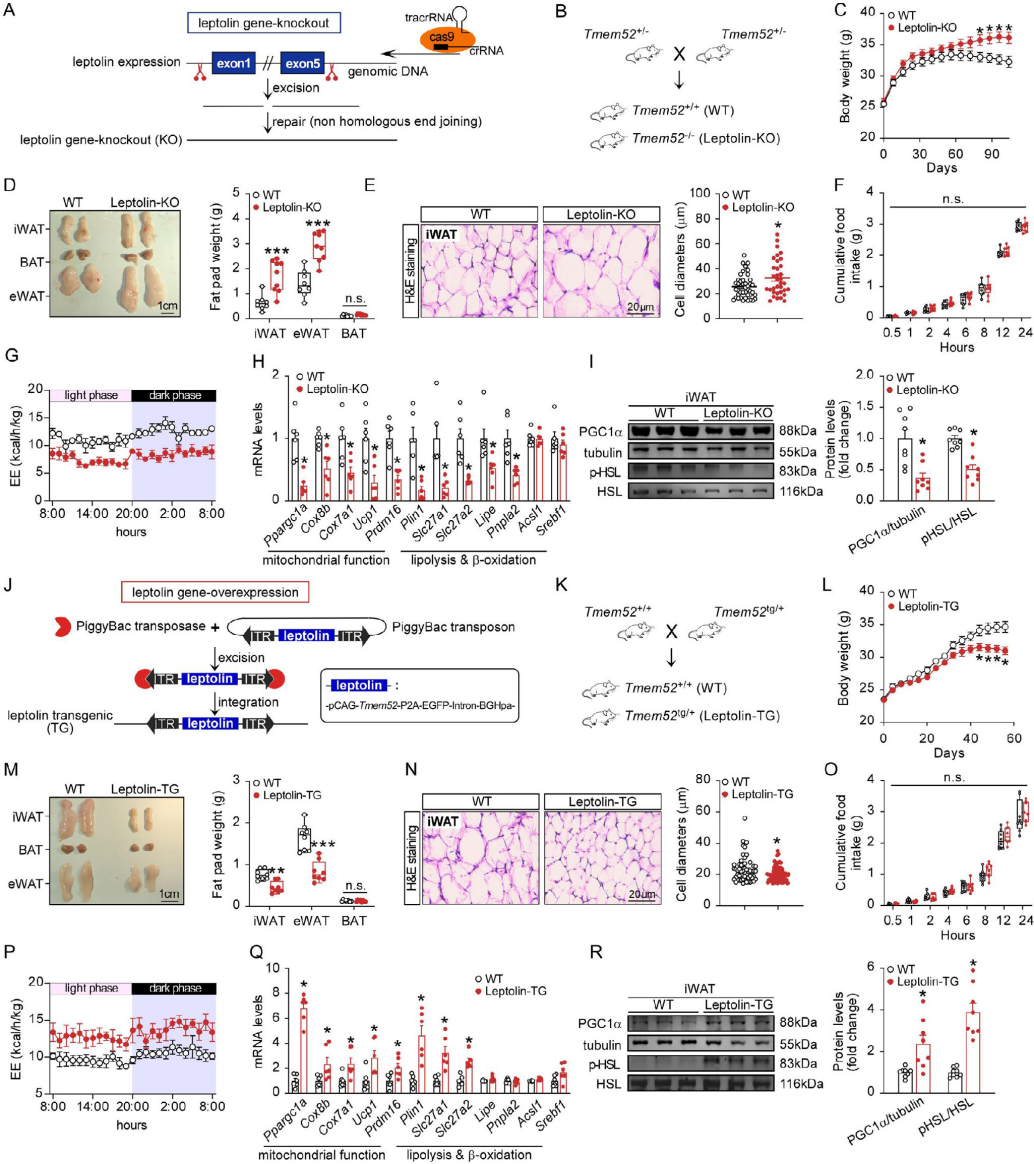
Leptolin deficiency increases the susceptibility to high‐fat diet (HFD)‐induced obesity, while leptolin gene overexpression prevents HFD‐induced obesity. (A) Schematic of the knockout strategy for *Tmem52* gene. (B) Breeding strategy for generation of *Tmem52* ^+/+^ mice (WT) and *Tmem52* ^−/−^ mice (leptolin‐KO). (C‐I) 8‐week‐old mice were fed a high‐fat‐diet, tissue harvest was performed at month 3. (C) Body weight; WT, *n* = 8, Leptolin‐KO, *n* = 9. (D) Representative images of fat pads (iWAT, eWAT, and BAT) and fat pad weight; WT, *n* = 8, Leptolin‐KO, *n* = 9. (E) Representative images of hematoxylin and eosin (H&E) staining of iWAT and the size profiling of adipocytes from iWAT; scale bar indicates 20 µm. (F) Cumulative food intake; WT, *n* = 8, Leptolin‐KO, *n* = 9. (G) Indirect calorimetry was performed to quantify EE of WT and leptolin‐KO mice during complete 24 h light‐dark cycles. WT, *n* = 4, Leptolin‐KO, *n* = 5. (H) Relative mRNA expression (fold change) of genes associated with mitochondrial function and fat mobilization in iWAT; *n*  =  6 per group. (I) Representative immunoblots of PGC1α, tubulin, p‐HSL^(S660)^ and HSL from iWAT, and the quantified ratio of PGC1α/tubulin and p‐HSL^(S660)^/HSL; *n*  =  8 per group. (J) Schematic of the overexpression strategy for *Tmem52* gene. (K) Breeding strategy for generation of wild type mice (WT) and leptolin gene‐overexpression mice (leptolin‐TG). (L‐R) 8‐week‐old mice were fed a high‐fat‐diet, tissue harvest was performed at month 2. (L) Body weight; WT, *n* = 9, Leptolin‐TG, *n* = 8. (M) Representative images of fat pads (iWAT, eWAT and BAT) and fat pad weight; n = 8 per group. (N) Representative images of H&E staining of iWAT and the size profiling of adipocytes from iWAT; scale bar indicates 20 µm. (O) Cumulative food intake; *n* = 8 per group. (P) Indirect calorimetry was performed to quantify EE of WT and leptolin‐TG mice during complete 24 h light‐dark cycles. WT, *n* = 4, Leptolin‐TG, *n* = 4. (Q) Relative mRNA expression (fold change) of genes associated with mitochondrial function and fat mobilization in iWAT; *n*  =  6 per group. (R) Representative immunoblots of PGC1α, tubulin, p‐HSL^(S660)^ and HSL from iWAT, and the quantified ratio of PGC1α/tubulin and p‐HSL^(S660)^/HSL; *n*  =  8 per group. Data are mean ± s.e.m.; student's t‐test was used for statistical analysis; ^*^ *p* < 0.05, ^**^ *p* < 0.01, ^***^ *p* < 0.001, n.s., not significant. See also Figures S9–S17.

Leptolin protein levels in iWAT were elevated (about 4‐fold) under the conditions of exercise and cold exposure (Figures S5B and S7A,B). Immunofluorescence analysis revealed distinct perinuclear and cell membrane localization patterns of leptolin protein in both adipocytes of WAT (Figure 2G) and 3T3‐L1 adipocytes (Figure S7C,D). Leptolin co‐distributed with vesicle‐associated membrane protein 2 (VAMP2) in WAT and adipocytes. Super‐resolution fluorescence‐assisted diffraction computational tomography (SR‐FACT) [28] detected leptolin‐EGFP signals within vesicles of live HEK293T cells transfected with a plasmid encoding leptolin (Figure S7E and Video S1). Intriguingly, mirroring previous studies [29, 30, 31], our confocal microscopy also revealed a membranous fluorescence pattern of leptolin in mature adipocytes, showing that leptolin has typical characteristics of a secretory protein.

Our in vivo experiments showed that leptolin mRNA and protein could be produced by adipocytes in iWAT (Figure S8A,B). Leptolin in cellular vesicles was remarkably increased under cold exposure (Figure S8B). Moreover, our in vitro experiments confirmed that leptolin could be produced by adipocytes (Figure S8C,D). Notably, we detected leptolin protein in the medium of 3T3‐L1 cells (Figure S8E). Interestingly, leptolin protein level was elevated under the condition of β3‐adrenergic activation (Figure S8D,E). Leptolin expression could be induced in mature adipocytes rather than in adipose progenitor cell (Figure S8C–E). These results verified that leptolin is an adipocyte‐derived adipokine. Adipocytic β3‐adrenergic signaling may be involved in the regulation of leptolin secretion (Figure S8F).

Further, we detected whether leptolin exists in mouse blood serum. Of note, quantitative analysis revealed a detectable level of circulating leptolin in the serum of mice (Figure 2H). Exercise or cold exposure could increase the levels of circulating leptolin (about 2‐fold) (Figure 2H). Western analysis demonstrated an absence of leptolin in serum samples of leptolin‐KO mice (Figure S9A,B). These observations demonstrate that leptolin may be an adipocyte‐derived secreted factor.

Pearson's correlation analysis of our murine WAT transcriptomic profiling data revealed a coordinated expression pattern between *Tmem52* and adipocytic secretory pathway components, particularly *Camk2a*, *Gorasp1*, *Syn2*, and *Vamp7* (Figure 2I), showing a potential role of CaMKII‐GRASP signaling [32] and type III unconventional protein secretory (UPS) pathway [33] in leptolin secretion. Correlation analysis also revealed an overlap between *Tmem52*‐positively related genes and mitochondrial/lipolytic pathways (*Ppargc1a*, *Cox7a1*, *Cox8b*, *Ucp1*, and *Slc27a2*) (Figure 2J), suggesting roles of leptolin in the regulation of mitochondrial function and lipolysis. Collectively, our findings indicate that the production of leptolin in WAT can be induced by exercise and cold exposure. Elevated circulating leptolin contributes to enhanced lipolysis, increased EE, and reduced adiposity (Figure S7F).

### 2.4 *Tmem52* Deficiency Reduces EE and Increases the Susceptibility to High‐Fat Diet (HFD)‐Induced Obesity

We further explore whether leptolin may reduce adiposity and promote energy expenditure (Figure 3A,B). The body weight of leptolin‐KO mice was 11.9% higher than that of controls at month 3 on a high‐fat diet (Figure 3C). The fat‐pad weight of iWAT and epididymal WAT (eWAT) was increased in leptolin‐KO mice, while the weight of brown adipose tissue (BAT) was not altered (Figure 3D). The size of adipocytes in iWAT of leptolin‐KO mice was 27.3% larger than that of controls (Figure 3E). At thermoneutrality, the body weight and fat mass of leptolin‐KO mice were also raised (Figure S10A–D). The cumulative food intake did not differ between groups (Figure 3F). A reduced whole‐body EE and an increased respiratory exchange ratio (RER) were observed in leptolin‐KO mice (Figure 3G and Figure S11A,B), but there was no change in the daily locomotor activity (Figure S11C). The gene expression associated with mitochondrial function (*Ppargc1a*, *Cox8b, Cox7a1, Ucp1*, and *Prdm16*) and lipolysis (*Plin1, Slc27a1, Slc27a2, Lipe*, and *Pnpla2*) were downregulated in iWAT of leptolin‐KO mice (Figure 3H). Protein levels of PGC1α, a key regulator for mitochondrial biogenesis, and p‐HSL, a canonical marker of lipolysis, were decreased in iWAT as compared with controls (Figure 3I). After 90 days on the high‐fat diet, the blood glucose levels of leptolin‐KO mice were higher than that of controls during the intraperitoneal glucose tolerance test (GTT) (Figure S12A–C); insulin, triglyceride and total cholesterol levels in serum of leptolin‐KO mice were higher than that of controls (Figures S12D and S13A–C). Together, leptolin deficiency suppresses the gene expression linked to mitochondrial function and lipolysis, reduces EE, and increases the susceptibility to HFD‐induced obesity, indicating effects of leptolin on lipid catabolism and energy expenditure.

### 2.5 Adipocyte‐Specific *Tmem52*‐Knockout in Inguinal WAT Dampens Cold Stress‐Induced Weight Loss

To specifically knockout *Tmem52* gene in adipocytes, we generated *Tmem52* ^flox/flox^ mice. We designed and produced an adeno‐associated virus (AAV8‐adipoq‐iCRE‐WPRE) vector to specifically knockout *Tmem52* gene in adipocytes of iWAT. We injected the AAVs into the bilateral iWAT fat pads of *Tmem52* ^flox/flox^ mice, and subjected these mice to cold stress (Figure S14A). We observed that leptolin protein levels were markedly decreased in iWAT and blood plasma under conditions of room temperature and cold exposure (Figure S14B,C). Notably, the cold‐induced reduction in both of fat‐pad weight and body weight was dampened in iWAT adipocyte‐specific *Tmem52* knockout mice (Figure S14D,E). Collectively, these results demonstrate that leptolin in adipocytes plays an important role in the regulation of adipose homeostasis.

### 2.6 Leptolin Gene‐Overexpression Elevates EE and Prevents HFD‐Induced Obesity

To further determine the effects of leptolin, we generated transgenic mice that over‐express leptolin (Figure 3J,K). There was an increased leptolin level in serum of leptolin‐TG mice, as compared with that of wild‐type controls (Figure S9A,B). We found that leptolin transgenic (TG) mice fed an HFD had a 10.6% reduction in body weight, as compared with controls after 2 months (Figure 3L). The fat‐pad weight of iWAT and eWAT were reduced (Figure 3M) and the size of adipocytes in iWAT of leptolin‐TG mice was 11.8% smaller than that of controls (Figure 3N). The food consumption was not altered (Figure 3O). Notably, leptolin‐TG mice had increased EE and decreased RER (Figure 3P and Figure S15A,B); locomotor activity was unaffected (Figure S15C). The mRNA levels of the genes involved in mitochondrial function (*Ppargc1a*, *Cox8b, Cox7a1, Ucp1*, and *Prdm16*) and lipolysis (*Plin1, Slc27a1*, and *Slc27a2*), and the protein levels of PGC1α and p‐HSL were enhanced in iWAT of leptolin‐TG mice (Figure 3Q,R). After 60 days on the HFD, leptolin‐TG mice exhibited a lowered blood glucose level during GTT and insulin tolerance test (ITT) (Figure S16A–C). The plasma insulin and triglyceride levels were reduced in leptolin‐TG mice (Figures S16D and S17A,B); the total cholesterol level in plasma was not changed (Figure S17C). Taken together, leptolin overexpression induces genes involved in mitochondrial function and lipolysis, elevates EE, and prevents the development of HFD‐induced obesity.

### 2.7 Leptolin Levels in Subcutaneous WAT and Blood Serum are Lowered in Obese Individuals and Increased in Athletes

**FIGURE 4:**
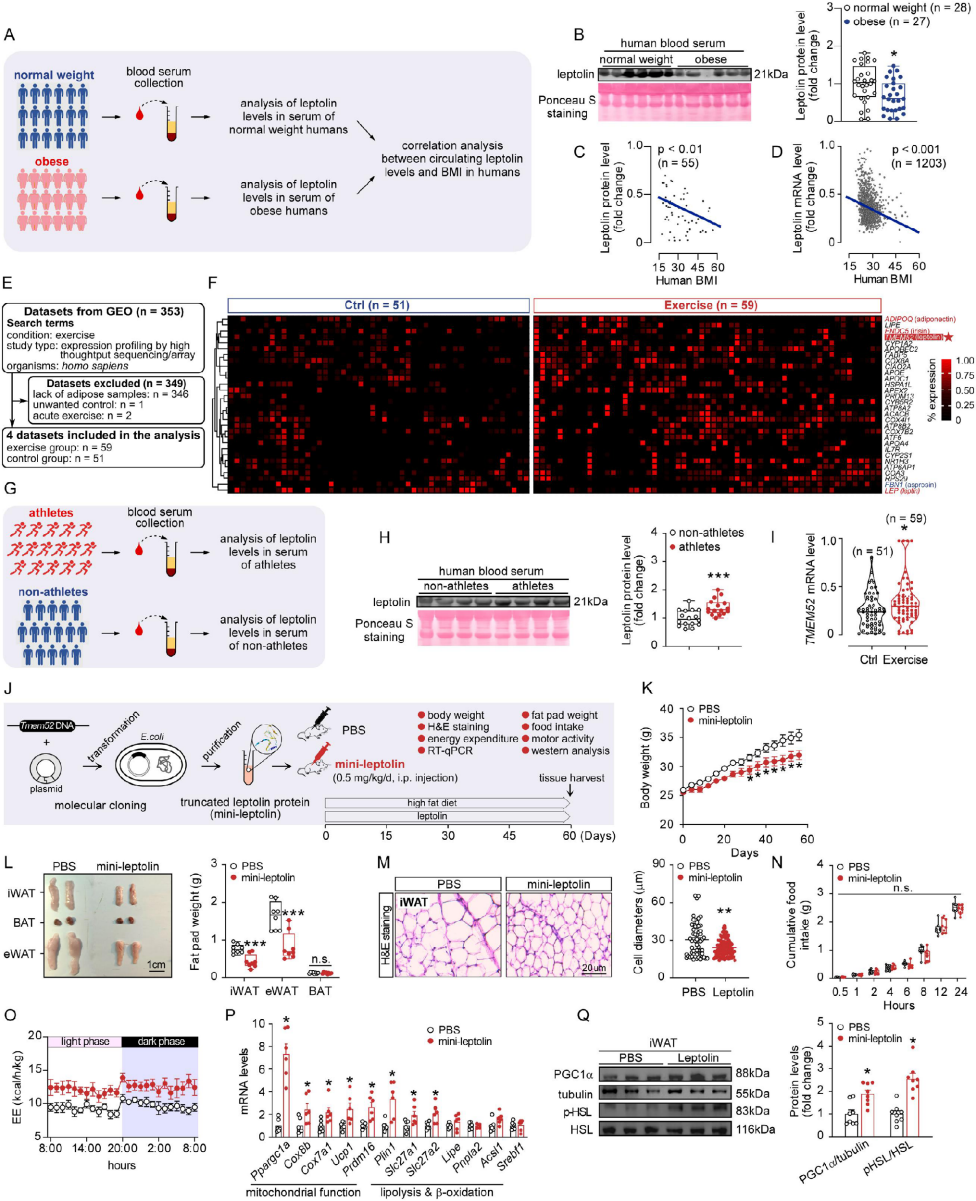
Leptolin administration enhances EE and prevents HFD‐induced obesity. (A) Schematic illustration of workflow for analysis of leptolin in the serum of normal weight and obese humans. (B) Representative immunoblots of leptolin and images of Ponceau S staining from serum of normal weight and obese humans, and the quantified ratio of leptolin/Ponceau S; normal weight, *n* = 28, obese, *n* = 27. (C) Scatter plots of BMI and leptolin protein levels in human serum, *n* = 55; the blue line shows correlation. (D) Scatter plots of BMI and *TMEM52* mRNA levels in sWAT of differently normalized large sample human datasets (*n* = 1203); the blue line shows correlation. (E) Schematic illustration of screening workflow of ctrl/exercise human sWAT datasets, 4 datasets are included in the analysis. (F) Heatmaps representing the selected 30 genes expression in human sWAT of exercise versus ctrl group. Ctrl, *n* = 51, Exercise, *n* = 59. (G) Schematic illustration of workflow for analysis of leptolin in serum of non‐athletes and athletes. (H) Representative immunoblots of leptolin and images of Ponceau S staining from serum of athletes and non‐athletes, and the quantified ratio of leptolin/Ponceau S; *n*  =  16 per group. (I) Analysis of *TMEM52* gene expression in sWAT of differently normalized Ctrl/Exercise human datasets. Ctrl, *n* = 51, Exercise, *n* = 59. (J) Schematic of production strategy for mini‐leptolin; and illustration of experiments: 8‐week‐old mice were fed a high‐fat‐diet, received PBS for 4 days as acclimation, then were intraperitoneally injected with mini‐leptolin or PBS daily for 2 months, tissue harvest was performed at month 2. (K) Body weight; *n* = 8 per group. (L) Representative images of fat pads (iWAT, eWAT, and BAT) and fat pad weight; *n* = 8 per group. (M) Representative images of H&E staining of iWAT and the size profiling of adipocytes from iWAT; scale bar indicates 20 µm. (N) Cumulative food intake; *n* = 8 per group. (O) Indirect calorimetry was performed to quantify EE of PBS and leptolin‐treated mice during complete 24 h light‐dark cycles. *n* = 5 per group. (P) Relative mRNA expression (fold change) of genes associated with mitochondrial function and fat mobilization in iWAT; *n*  =  6 per group. (Q) Representative immunoblots of PGC1α, tubulin, p‐HSL^(S660)^, and HSL from iWAT, and the quantified ratio of PGC1α/tubulin and p‐HSL^(S660)^/HSL; *n*  =  8 per group. Data are mean ± s.e.m.; student's t‐test was used for statistical analysis; ^*^ *p* < 0.05, ^**^ *p* < 0.01, ^***^ *p* < 0.001, n.s., not significant. See also Figures S18–S25.

We detected leptolin in serum of normal weight and obese humans (Figure S18A and Figure 4A). In obese (BMI ≥ 28) individuals, a reduction of about 33% in leptolin levels was detected in serum compared with the normal weight individuals (Figure 4B). A negative correlation between circulating leptolin levels and BMI in humans was found (Figure 4C). Notably, *TMEM52* mRNA levels in sWAT were also negatively correlated with body mass index (BMI) of humans (Figure S18B and Figure 4D). A positive correlation between sWAT mRNA levels of *TMEM52* and the lipolysis‐promoting genes (*PPARGC1A*, *COX8B, LIPE, SLC27A1*, and *SLC27A2*) was found (Figure S18C).

Notably, elevated *TMEM52* mRNA levels in sWAT of humans with exercise were observed, as compared with that of non‐exercised humans (Figure 4E,F). Thus, we then detected leptolin in human blood serum of athletes and non‐athletes (Figure S18D and Figure 4G). In adult athletes undergoing supervised endurance exercise training, a 38.4% increase in the circulating leptolin levels were observed compared with the non‐athletes (Figure 4H). Intriguingly, *TMEM52* mRNA levels in sWAT were also increased under conditions of exercise (Figure 4I). *TMEM52* mRNA levels were positively correlated with *PPARGC1A*, *COX8B, HSL, SLC27A1*, and *SLC27A2* in sWAT of these non‐exercised and exercised humans (Figure S18E).

Taken together, *TMEM52* mRNA levels are decreased in sWAT of obese individuals and increased by exercise; leptolin protein is present in human serum, which is lowered in obese individuals and heightened in athletes. These results suggest a therapeutic potential of leptolin for obesity treatment in humans.

### 2.8 Leptolin Administration Enhances EE and Prevents HFD‐Induced Obesity

To evaluate the therapeutic potential of leptolin in treating obesity, we cloned and produced a truncated (68‐196 aa) mouse leptolin protein (mini‐leptolin) for intraperitoneal (I.P.) injection (Figure 4J). The dose‐effect study showed that mini‐leptolin injection at the dosage levels of 0.5 or 5 mg/kg/d for 2 months effectively reduced body weight and elevated EE in HFD‐fed mice, without altering daily food intake (Figure S19A–E). The mini‐leptolin‐treated mice did not display adverse reactions, and there was no apparent toxicity in major organ systems. Thus, mini‐leptolin I.P. injection (0.5 mg/kg/d, once daily for 2 months) was selected to study its anti‐obesity effects. We found that mini‐leptolin treatment markedly reduced body weight in HFD‐fed mice, equivalent to a total weight loss of 10.8% at month 2 (Figure 4K). It also led to a reduced weight of iWAT and eWAT (Figure 4L), and a 20.5% smaller size of adipocytes in iWAT (Figure 4M). Cumulative food intake was not altered (Figure 4N). Mini‐leptolin enhanced whole‐body EE and decreased RER (Figure 4O and Figure S20A,B), and did not change the motor activity (Figure S20C). Infrared thermal imaging analysis showed that leptolin markedly elevated the body surface temperature (Figure S20D). Mini‐leptolin increased gene expression associated with mitochondrial function (*Ppargc1a*, *Cox8b, Cox7a1, Ucp1*, and *Prdm16*) and lipolysis (*Plin1, Slc27a1*, and *Slc27a2*), and elevated protein levels of PGC1α and p‐HSL in iWAT (Figure 4P,Q). Mini‐leptolin also induced the expression of these genes in eWAT and BAT (Figure S21A,B). After 60 days of mini‐leptolin treatment, improved blood glucose levels during GTT and ITT were detected (Figure S22A–C). The plasma insulin and triglyceride levels were lowered (Figures S22D and S23A,B). The plasma total cholesterol level did not differ between groups (Figure S23C). Together, mini‐leptolin administration promotes gene expression relative to mitochondrial function and lipolysis, enhances EE, and reduces weight gain.

Furthermore, we treated adipocytes with recombinant leptolin in vitro. We did not observe remarkable changes of the adipocytic morphology. Further, adipocyte size and lipid droplet dynamics (BODIPY and Oil Red O assays) were not changed (Figure S24A–C). Expression of the genes associated with mitochondrial function and lipolysis were unaltered (Figure S24D). These observations suggest that leptolin may exert its functional effects on adipocytes via other ways. Notably, we found that leptolin treatment elevated gene expression associated with neural activity (*Adrb3*, *Dio2*) in iWAT of mice (Figure S24E,F). In vivo imaging (IVIS SPECTRUM) results showed that the hypothalamus may be one of target tissues of leptolin (Figure S24G). Moreover, we observed that leptolin intraperitoneal (i.p.) and intracerebroventricular (i.c.v.) injection reduced fat pad weight and adipocyte size, and elevated expression of genes associated with mitochondrial function and lipolysis (Figure S25A–H). Intriguingly, we found that the effects of both peripheral (Figure S25A–D) or central (Figure S25E–H) administration of leptolin were dampened in sympathetically‐denervated iWAT, but not in contralateral sham‐operated iWAT. These new findings suggest that leptolin is likely to exert its pronounced effects in driving lipolysis and reducing adiposity via neuro‐endocrine mechanisms (Figure S24H).

## 3 Discussion

We detected the existence of leptolin protein in the blood serum of humans and mice, characterizing leptolin as a secreted protein [10, 34]. We observed that leptolin mRNA and protein levels in WATs, but not in other tissues or organs, were elevated in the conditions of physical exercise and cold exposure. In situ hybridization revealed that mRNA encoding leptolin was abundant in adipocytes. Leptolin protein was transported by secretory vesicles in adipocytes, revealing that it is an adipocyte‐derived hormone, or adipokine.

In humans, leptolin also could be detected in sWAT and blood serum. Meta‐analysis of global collection of human data showed that *TMEM52* mRNA levels were lowered in sWAT of obese individuals and elevated in sWAT of athletes. Of note, our experimental data showed decreased levels of leptolin protein in blood serum of obese individuals and raised levels of leptolin in blood serum of athletes. Human leptolin mRNA levels in sWAT and circulating leptolin protein levels were negatively associated with BMI, which is a feature of other anti‐obesity adipokines such as adiponectin [35, 36]. These findings uncover that leptolin may be a potential anti‐obesity adipokine, and it may have the capacity to improve metabolic status.

Leptolin promoted fat utilization, elevated EE, and prevented obesity. We observed that *Tmem52* gene‐overexpression mice showed elevated lipolysis, enhanced EE, and obesity‐resistant phenotypes. Conversely, *Tmem52* gene‐knockout mice exhibited adverse phenotypes. Treatment of exogenous leptolin promoted fat mobilization, increased EE, and led to a total weight loss of 10.8% at month 2 in HFD‐induced obese mice. Stimulation of fat utilization and elevation of EE are important therapeutic strategies for obesity [3, 37, 38]. Leptin, a canonical adipokine, can promote lipolysis and increase basal metabolic rate (BMR) [5, 39]. Irisin, an adipokine/myokine, can act on adipocytes to trigger lipolysis and elevate EE [10, 40, 41]. These findings suggest that enhancement of lipolysis and catabolic metabolism and the elevation of EE may underlie the anti‐obesity effects of leptolin.

Adipokines are crucial in the regulation of lipid catabolism [42]. Dysfunction of adipokines contributes to obesity and related complications [42]. Adipokines are potential therapeutic agents in obesity [43]. For instance, leptin deficiency results in severe obesity [44]; leptin replace therapy promotes lipolysis, elevates EE, and inhibits food intake to reduce body weight [5, 13]. Adiponectin levels in plasma are lowered in obesity [36]; Adiponectin treatment enhances EE, reduces food intake, and decreases body weight [8, 9]. Administration of irisin activates white adipocytes and elevates EE to reduce body weight [10]. In this study, we detected a decreased level of leptolin in the blood serum of obese patients. Moreover, leptolin deficiency augmented fat‐pad weights and body weight. Notably, leptolin administration stimulated lipolysis, enhanced EE and reduced body weight. These findings uncover that leptolin may hold promise in obesity treatment. Exploring the clinical utility of leptolin may shed new light on the therapy for obesity and related disorders.

Obesity is primarily the result of dysregulation of energy intake and energy expenditure [3, 45]. To date, the anti‐obesity drugs are usually associated with appetite suppression [46, 47]. Semaglutide mimics the hormone glucagon‐like peptide 1 (GLP‐1) to inhibit appetite and reduce food intake, thereby inducing weight loss [48]. These appetite‐inhibiting drugs have short‐term side effects including nausea, vomiting and diarrhea; and raise the risk of cardiovascular disease, osteoporosis and other disorders [46, 49]. Unlike these appetite‐inhibiting drugs, leptolin administration elevated EE and reduced weight loss, but did not affect food intake. The anti‐obesity drugs, such as semaglutide and tirzepatide, may lead to a loss of lean body mass (including muscle and bone) [46, 50]. Exercise can improve metabolic status and lead to a healthier proportion of lean‐to‐total body mass [51]. Thus, the “exerkine” leptolin may have the potential to improve metabolic status, reduce body weight, and fat pad weight without reducing lean body mass. In the present study, we did not observe apparent toxicity in muscle, bone, and other major organs of the leptolin‐treated mice. Of note, *Tmem52* gene knockout or overexpression, or leptolin administration for 2 months did not alter the motor activity of mice, reflecting that leptolin did not affect food‐seeking behavior. Overall, leptolin may be a promising anti‐obesity agent with safety, and leptolin action may recapitulate the benefits of exercise on metabolic health.

Human and mouse leptolin protein are 77% identical. Both human and mouse leptolin expression was inversely correlated with obesity. Functions of adipokines, such as leptin, adiponectin, FGF21 and resistin, are mediated by specific receptor‐ligand interactions [5, 9, 52, 53]. Identification of leptolin receptor is crucial for deciphering the function of leptolin [54]. To date, we have identified three candidate receptors (*Paqr4*, *Armc10* and *Gpr151*) by means of receptor searching algorithms and bioinformatic prediction. Identification and study of leptolin receptor may deepen the understanding of leptolin actions.

## 4 Conclusion

In summary, our studies in mice and humans identified a novel anti‐obesity adipokine, the leptolin, encoded by *TMEM52* gene. Leptolin had a capacity to improve metabolic status and might serve as a new therapeutic agent for obesity and metabolic disorders.

## 5 Methods

### 5.1 Mice and Animal Care

Mice were housed at 22 ± 1°C with a 12‐h light/dark cycle. Standard mouse chow and water were freely available except where otherwise indicated. All procedures were approved by the Institutional Care and Use Committee of the Peking University Health Science Center (approval number: **LA2019340**). All animals were sex‐ and age‐matched, and littermates were used, as indicated in the figures. Animals were allocated to their experimental group according to their genotypes. *Tmem52* ^+/+^ and *Tmem52* ^−/−^ mice were generated by crossing heterozygous *Tmem52* ^+/−^ mice. Wild type (WT) and *Tmem52* gene‐overexpression (leptolin‐TG) mice were generated by crossing the C57BL/6N mice and leptolin‐TG mice. C57BL/6N male mice were obtained from the Department of Laboratory Animal Science of Peking University Health Science Center. For diet‐induced obesity studies, mice were placed on the 60 kcal% HFD (D12492i; Research Diets) at the age of 8 weeks. For thermoneutral studies, mice were housed at 30°C in a light‐controlled climatic chamber. During all procedures of experiments, the number of animals and their suffering by treatments were minimized.

### 5.2 Human Subjects

These nonobese/obese human subjects were obtained from the Peking University Third Hospital (approval number: **202‐226‐02**). Informed consent was obtained from all patients or their parents/ guardians. All data were kept confidential and processed anonymously. The research encompassed 55 samples of human blood serum, procured from 27 obese individuals (BMI ≥ 28 kg/m^2^) and 28 nonobese individuals (18< BMI < 28 kg/m^2^), with matching age and sex. Weight was measured before blood collection. The nonathletes/athletes human subjects were obtained from the Beijing Sport University (approval number: **2018018H**). Informed consent was obtained from all participants. All data were kept confidential and processed anonymously. The research encompassed 32 samples of human blood serum, procured from 16 athletes and 16 nonathletes with matching age and sex. All of the participants displayed apparent health, without any record of excessive alcohol consumption.

### 5.3 Establishment of Fat Burning Models

Running exercise was carried out as previously described; briefly, 10‐week‐old mice were housed individually with ad libitum food and water; one week before the experiment, mice were adapted on a motor treadmill at a speed of 10 m/min for 10 min on the first day and increased the exercise time by 10 min each day until it reached 60 min per day; then, this exercise strength for mice was maintained for another two weeks. Swimming exercise was carried out as described; briefly, 10‐week‐old mice were trained to swim for 10 min in warm (30°C) water and twice a day; the swimming time were increased by 10 min each day until reaching 90 min per day; then, this exercise strength for mice was maintained for another two weeks; we closely checked the mice to avoid anoxia and kept the water temperature at 30°C to avoid hypothermia; after each test session, mice were towel‐dried and placed back in their home cage. Cold exposure was conducted according to a previously established program; in brief, 10‐week‐old mice were housed individually with ad libitum food and water; mice were placed at 4°C for seven days before sacrificing.

### 5.4 Whole Transcriptome Sequencing and Analysis

Total RNA was extracted from iWAT of Ctrl, Run, Swim, or Cold group mice using TRIzol reagent (TransGen Biotech). The quality of the RNA was determined with the NanoDrop 5500 (Thermo Fisher Scientific). For library preparation, 3 µg of total RNA/sample was used. Sequencing libraries were generated with NEBNext Ultra RNA Library Prep Kit for Illumina (New England Biolabs, Ipswich, MA). RNA molecules were selected using poly‐T oligo‐attached magnetic beads, fragmented, and reverse transcribed with the Elute, Prime, Fragment Mix. Then, end repair, A‐tailing, adaptor ligation, and library enrichment were performed according to the manufacturer's instructions. RNA libraries were assessed for quality using the Agilent 2100 Bioanalyzer. The clustering of the index‐coded samples was performed on a cBot Cluster Generation System using TruSeq PE Cluster Kit v3‐cBot‐HS (Illumina). Then, the RNA libraries were sequenced as 100‐bp/50‐bp paired‐end runs on an Illumina HiSeq 2000/2500 platform. Differential expression analysis of two conditions/groups (two biological replicates per condition) was performed using the DESeq R package (1.10.1). The resulting *p* values were adjusted using the Benjamini‐Hochberg approach for controlling the false discovery rate. Genes with an adjusted *p* value <0.05 found by DESeq were assigned as differentially expressed. Gene Ontology (GO) enrichment analysis of differentially expressed genes (DEGs) was performed using the GOseq R package, in which gene length bias was corrected. Samples were measured in Novogene Bioinformatics Technology Co., Ltd.

### 5.5 Generation of Leptolin‐KO Mice

For the generation of *Tmem52* ^−/−^ (leptolin‐KO) mice, Cas9/gRNAs were microinjected into the fertilized eggs. Injected eggs were implanted into surrogate C57BL/6N mothers to obtain offspring. Genotyping was performed to identify those harboring *Tmem52* deficiency. Heterozygous mice were used to generate homozygous *Tmem52* ^+/+^ and *Tmem52* ^−/−^ mice. Primers for genotyping are as follows: *Tmem52*‐WT forward: 5′‐ AGA AGC AGG CAT TCC AGG TTG AG ‐3′, reverse: 5′‐ AGA GGC AGC AGC AGC AGG AT ‐3′, *Tmem52*‐KO forward: 5′‐ AGC AGG CAT TCC AGG TTG AG ‐3′, reverse: 5′‐ CAG TCC AGG TCT GTG GTC AC ‐3′.

### 5.6 Generation of Leptolin‐TG Mice

For the generation of pCAG‐*Tmem52*‐P2A‐EGFP transgenic mice, a vector encoding leptolin‐P2A‐EGFP under the control of cytomegalovirus (CMV) enhancer fused to the chicken beta‐actin promoter (CAG promoter) was obtained. The plasmid was microinjected into the pronucleus of fertilized eggs. Injected eggs were implanted into surrogate C57BL/6N mothers to obtain offspring. Genotyping was performed to identify those carrying the *Tmem52* transgene. Protein levels of exogenous leptolin in serum was approximately doubled in leptolin‐TG mice. Primers for genotyping are as follows: *Tmem52*‐TG forward: 5′‐ GCC ATA CTC CTG ATG CTT TTG T ‐3′, reverse: 5′‐ AGT TCA CCT TGA TGC CGT TCT ‐3′.

### 5.7 Production of Mini‐Leptolin Protein for Injection

Mouse *Tmem52* cDNA was cloned and subsequently sub‐cloned into a pET‐22B vector for expression in *Escherichia coli*. The fusion protein that was expressed in *E. coli* is 135 amino acids long comprising of a 6‐amino‐acid His tag on the N terminus and a 129‐amino‐acid leptolin fragment (68‐196 amino acids). The His‐leptolin was isolated from *E. coli* and allowed to bind to Ni‐NTA His‐Bind column. After extensive washing of the column to remove contaminating proteins, His‐leptolin were eluted from the column using a 150‐mm imidazole buffer. Then the His tag was cut, and the leptolin protein was further purified using size‐exclusion columns and polymyxin B‐based endotoxin‐depletion columns (Detoxi‐Gel Endotoxin Removing Gel by Thermo Scientific), to bring the final endotoxin concentration equal to or below 2 EU/mL. The buffer was exchanged into a PBS buffer.

### 5.8 Administration of Mini‐Leptolin

Administration of mini‐leptolin was carried out by i.p. injection. For i.p. treatment, mice received 100 µL of vehicle (PBS) for 4 days as acclimation before leptolin treatment. Then, mini‐leptolin was dissolved in PBS (100 µL; 0.005, 0.05, 0.5, and 5 mg/kg) and administered to mice once a day for 2 months; vehicle groups received 100 µL of PBS during the course of the experiments. All treatments were performed within 90 min of the dark cycle.

### 5.9 AAVs Production and Vectors Injection

AAV8‐adipoq‐iCRE‐WPRE vectors were constructed in OBiO Technology. Vectors injection procedures were performed as previously described. Briefly, 8‐week‐old male *Tmem52* ^flox/flox^ mice were injected at multiple sites of the inguinal WAT pads (Virus diluted in sterile PBS: 1 × 10^10^ PFU/100 µL). Two weeks after vectors injections, mice were subjected to cold stress for 8 days. Body weights were monitored throughout the duration of the experiment.

### 5.10 Real‐Time Quantitative PCR

RNA was extracted from the tissues using TransZol Up reagent (ET111‐01, TransGen Biotech), followed by reverse transcription using a reverse transcription kit (AT311‐03, TransGen Biotech). cDNAs were processed for real‐time PCR using SYBR Green mix (AQ141‐04, TransGen Biotech) with specific primers on a StepOnePlus real‐time PCR System (Roche). All data were normalized with *Gapdh*.

### 5.11 RNAscope

Inguinal WATs were collected from mice, frozen in OCT compound (Sakura Finetek), and immediately followed by preservation at ‐80°C according to standard procedure. Tissue sections (SuperFrost Plus microslides) of 10–25 µm thickness were taken on a cryostat. The sections were immediately fixed in 4% paraformaldehyde solution for 15 min. Then, the sections were incubated sequentially with 50% ethyl alcohol for 5 min, 70% ethyl alcohol for 5 min, and 100% ethyl alcohol two times for 5 min and allowed to air dry on slides for 5 min. Sections were incubated with RNAscope Hydrogen Peroxide for 2 h at 40°C, and were washed in 1X Wash Buffer two times for 2 min at room temperature (RT). Sections were incubated with RNAscope 2.5 AMP 1 for 30 min at 40°C, with RNAscope 2.5 AMP 2 for 15 min at 40°C, with RNAscope 2.5 AMP 3 for 30 min at 40°C, with RNAscope 2.5 AMP 4 for 15 min at 40°C, with RNAscope 2.5 Amp 5‐RED for 30 min at RT, and with RNAscope 2.5 Amp 6‐RED for 15 min at RT. Then sections were incubated with RNAscope RED working solution for 10 min at RT, and were washed in distilled water two times. Sections were incubated with 50% Hematoxylin staining solution for 2 min at RT and then were incubated with 0.02% Ammonia water. Sections were mounted with VectaMont medium and analyzed on a microscope (Histology Facility of the Department of Anatomy, Histology and Embryology, Peking University).

### 5.12 Total Protein Extraction and Western Blotting

Proteins were extracted from iWAT, eWAT, BAT or blood serum using a RIPA lysis buffer containing 0.5% NP‐40, 0.1% sodium deoxycholate, 150 mm NaCl, 50 mm Tris–HCl (pH 7.4), phosphatase inhibitors (B15002, Bimake), and protease inhibitor cocktail (B14002, Bimake). Following 5 min of homogenization, lysates were centrifuged at 11250 r for 15 min at 4°C. Supernatants from the fat pads were used as protein extracts. The concentration of each sample was calculated by the BCA method and an equal amount of protein from each sample was added an equal amount of protein loading buffer, this buffer should contain 5% β‐mercaptoethanol (vol/vol) or 20 mmol/L TCEP (for western blotting of leptolin), and denatured by boiling at 100°C for 5 min. Equal amounts of proteins were separated by 10% SDS‐PAGE, transferred to NC membranes. The membranes were blocked for 2 h in 5% skim milk. The membranes were then incubated with the primary antibody in 5% BSA‐TSBT at 4°C. After overnight incubation, the membrane was washed three times in TBST for 15 min, followed by incubation with a secondary antibody in TBST with 5% skim milk for 2 h at room temperature. Following three cycles of 15 min washes with 1× TBST, the membranes were developed using a chemiluminescence assay. Intensities of the protein bands were quantified by ImageJ software. The antibodies used in this study include Anti‐PGC1α antibody (Bioss, bs‐1832R), Anti‐pHSL^(S660)^ antibody (Bioss, bs‐3358R), Anti‐HSL antibody (Bioss, bs‐0455R), Anti‐α/β‐Tubulin antibody (Cell Signaling Technology, 2148S), Anti‐Leptolin antibody (designed by us and produced by SinoBiological), Anti‐Rabbit IgG(H+L) (Biodragon, BF03008), and Anti‐Mouse IgG(H+L) (Biodragon, BF03001).

### 5.13 Immunofluorescence

Inguinal WATs were collected from mice and immediately fixed in 4% paraformaldehyde solution for 48 h. Then, the samples were incubated sequentially with 20% sucrose and 30% sucrose in PBS for 2 d and frozen in OCT compound (Sakura Finetek). Tissue sections of 10–25 µm thickness were taken on a cryostat and allowed to air dry on slides, followed by processing or preservation at −80°C according to standard procedure. 3T3L1 cells were placed and cultured in confocal dishes and fixed in 4% paraformaldehyde solution for 30 min. Frozen sections of tissues or confocal dishes of cells were subjected to leptolin or VAMP2 staining. Sections or dishes were washed in PBS for 10 min, followed by incubation in blocking solution (10% normal goat serum, 0.2% Triton X‐100, 2% BSA, and PBS) for at least 1 h at room temperature. Primary antibodies were applied in blocking solution and incubated overnight at 4°C. Sections or dishes were washed at least three times with 5‐min incubations in PBS plus 0.2% Triton X‐100. Then, an Alexa Fluor 488–conjugated secondary antibody (1:200) or Alexa Fluor 594–conjugated secondary antibody (1:200) was applied in blocking solution and incubated at room temperature for 2 h, followed by five washes with PBS plus 0.2% Triton X‐100, and nuclei were stained with DAPI. Sections or dishes were mounted with VECTASHIELD medium (Vector Laboratories) and analyzed on a microscope (Histology Facility of the Department of Anatomy, Histology and Embryology, Peking University). The antibodies used in this study include Anti‐Leptolin antibody (designed by us and produced by SinoBiological), Anti‐VAMP2 antibody (Proteintech, 67822‐1‐lg), Anti‐Rabbit IgG/Alexa Fluor 488 (Abcam, ab150108), and Anti‐Mouse IgG/Alexa Fluor 594 (Abcam, ab150108).

### 5.14 Hematoxylin and Eosin Staining

Animals were sacrificed and fats were immediately dissected and fixed in 4% paraformaldehyde solution for 48 h followed by cryopreservation in 25% sucrose solution (wt/vol) overnight and subsequent freezing in OCT compound (Tissue‐Tek). Samples were stored in optimal cutting temperature compound (OCT) for frozen. Samples were sectioned, and H&E stained. The cell size was calculated by Image J.

### 5.15 Metabolic Chamber

Indirect calorimetry recording was performed using an indirect open‐circuit calorimeter Oxylet Physiocage System (LE1305 Physiocage 00, LE405 O_2_/CO_2_ Analyzer, and LE400 Air Supply and Swithching; Panlab, Cornellà, Spain). Room air flowed through each chamber at a rate of 450 mL/min. The mice were placed in metabolic chambers with fresh food and water provided every day to acclimate for 24 h. Before dark cycle, the authors started to monitor the oxygen consumption (VO_2_), carbon dioxide production (VCO_2_), respiratory‐exchange‐ratio (RER), energy expenditure (EE), and motor activity for 24 h. The O_2_ and CO_2_ levels were measured during 3‐min sampling periods every 30 min, and data were analyzed with METABOLISM software (v2.2.01). Locomotor activity were measured using a two‐dimensional infrared light beam. The VO_2_ and VCO_2_ were expressed in milliliters per minute per kilogram. The respiratory exchange ratio (RER) was determined by the ratio VCO_2_/VO_2_. The EE was calculated with the Weir equation and expressed in kcal/h/kg. The mean values for RER, EE, and motor activity of the dark cycle and light cycle were compared for each group.

### 5.16 Insulin and Glucose Tolerance Tests

For insulin and glucose tolerance tests (ITT and GTT, respectively), mice were fasted for 4 and 16 h, respectively. Mice were injected intraperitoneally with bovine insulin (Sigma, 0.5 units/kg) or with 20% glucose (2.0 g/kg). Blood samples were taken at 0, 30, 60, and 120 min.

### 5.17 Plasma Parameter Assays

Whole blood was collected by cardiac puncture and transferred to ice‐cold EP tubes. The tubes were centrifuged at 2,000 g for 30 min at 4°C and stored at −80°C. The serum was used for Western blot and measurement of insulin, TG, and CHO levels. Insulin, triglycerides and cholesterol reagent kits were purchased from Biosino Bio‐Technology and Science Inc.

### 5.18 Body Composition Analysis

Body composition analysis was performed using EchoMRI device (Echo Medical Systems, Houston, TX) to assess fat and lean mass of the mice.

### 5.19 Cell Maintenance and Preparation

Human 293T cells were cultured in high‐glucose DMEM (GIBCO, 11965092) supplemented with 10% fetal bovine serum (FBS) (GIBCO) and 1% 100 mM sodium pyruvate solution (Sigma–Aldrich, S8636) in an incubator at 37°C with 5% CO_2_. 293T cells were transfected with a leptolin‐EGFP vector. Transfections were performed using Lipofectamine 2000 (Thermo Fisher Scientific, 11668019) according to the manufacturer's instructions. Images or videos of cells were obtained using SR fluorescence‐assisted diffraction computational tomography 24–36 h after transfection. 3T3L1 cells were cultured in high‐glucose DMEM (GIBCO, 11965092) supplemented with 10% fetal bovine serum (FBS) (GIBCO) and 1% 100 mm sodium pyruvate solution (Sigma–Aldrich, S8636) in an incubator at 37°C with 5% CO_2_. For preparation of differentiated 3T3L1 cells, 3T3L1 cells were cultured in high‐glucose DMEM (GIBCO, 11965092) supplemented with 10% fetal bovine serum (FBS) (GIBCO), 0.5 mm IBMX, 1 µm insulin and 0.5 µm dexamethasone. For β3‐adrenergic activation, 3T3L1 cells were cultured with Cl316,243.

### 5.20 Human and Mouse Datasets

4 Ctrl/Exercise human sWAT datasets (GSE43471, GSE115645, GSE116801, GSE159809) and 2 large sample human sWAT datasets with BMI information (GSE135134, GSE70353) were identified, log2‐transformation was applied as needed. To control for the effects of known and hidden covariates in each of the datasets, we used R/sva package to adjust expression data for known factor covariates with the combat function and then estimate surrogates for hidden covariates with the sva function. Within each dataset, the expression values were further standardized to µ  =  0 and σ  =  1. Scatter plots of BMI, expression levels of *TMEM52* and genes associated with mitochondrial function and fat mobilization were displayed, and correlation analysis was performed. 16 Normal weight/Obese human sWAT datasets (GSE141432, GSE166047, GSE165932, GSE152991, GSE159924, GSE24883, GSE44000, GSE55200, GSE59034, GSE88837, GSE156906, GSE48964, GSE205668, GSE29718, GSE133099, GSE94752) were identified, log2‐transformation was applied as needed, and meta‐analysis of *TMEM52* expression levels in these datasets was performed. A total of 2 Run/Ctrl mouse iWAT datasets (GSE199429, GSE183239), 2 Cold/Ctrl mouse iWAT dataset (GSE140259, GSE212349), 1 mechanical stress related dataset (GSE186208), and 2 Fast/Fed mouse iWAT datasets (GSE46495, GSE154611) were identified, log2‐transformation was applied as needed.

### 5.21 Statistical Analysis

Where indicated, data were expressed as mean ± standard error of means (SEM). Statistical significance was determined using Student t test (two‐tailed) or one‐way ANOVA. All data were analyzed using the appropriate statistical analysis methods with SPSS software (Windows version 26, IBM Analytics) or GraphPad Prism (Windows version 8.0, GraphPad Software). Significance was accepted at ^*^ *p* < 0.05, ^**^ *p* < 0.01, or ^***^ *p* < 0.001. Sample sizes (n), statistical tests and p values are indicated in each figure legend.

## Author Contributions

J.L. performed the experiment, analyzed the data, made the figures, did the literature search and wrote the paper. B.W., Z.S., X.H., M.H., Y.Z., Y.H., D.L., W.Z., L.Q., K.W., Y.L., Y.Y., S.Y., X.H., T.Y., and J.L. participated in the study. R.Z. conceived the study, designed experiments, and wrote and edited the paper.

## Funding

This work was supported by grants from the Noncommunicable Chronic Diseases‐National Science and Technology Major Project (No. 2024ZD0530200 and 2024ZD0530201; R.Z.), the National Natural Science Foundation of China (No. 81471064 and 81670779 and 81870590 and 82170864; R.Z.), the National Key Research and Development Program of China (No. 2017YFC1700402; R.Z.), the Beijing Municipal Natural Science Foundation (No. 7162097 and H2018206641; R.Z.), the Peking University Research Foundation (No. BMU20140366; R.Z.), the Scientific Project of Beijing Life Science Academy (No. 2023300CB0100; R.Z.), and the Clinical Medicine Plus X—Young Scholars Project of Peking University (No. PKU2024LCXQ009; J.L.).

## Conflicts of Interest

The authors declare no conflicts of interest.

## Supporting information




**Supporting File 1**: advs75800‐sup‐0001‐SuppMat.docx.

**Supporting File 2**: advs75800‐sup‐0002‐Video1.avi.

## Data Availability

The data that support the findings of this study are available on request from the corresponding author. The data are not publicly available due to privacy or ethical restrictions.
