# Supplementary material for: Adipocyte‐Derived Leptolin Enhances Energy Expenditure and Prevents Obesity
Source: Adv Sci (Weinh). 2026 May 25;13(46):e16081. doi: 10.1002/advs.202516081 (PMC13335978; doi:10.1002/advs.202516081)
Supplement: Supplementary file 1 [file ADVS-13-e16081-s002.docx]

**Supplementary information**

**Adipocyte-derived leptolin enhances**

**energy expenditure and prevents obesity**

**Figure-S1**

**Figure-S2**

**Figure-S3**

**Figure-S4**

**Figure-S5**

**Figure-S6**

**Figure-S7**

**Figure-S8**

**Figure-S9**

**Figure-S10**

**Figure-S11**

**Figure-S12**

**Figure-S13**

**Figure-S14**

**Figure-S15**

**Figure-S16**

**Figure-S17**

**Figure-S18**

**Figure-S19**

**Figure-S20**

**Figure-S21**

**Figure-S22**

**Figure-S23**

**Figure-S24**

**Figure-S25**

**Figure-S26**

**Supplemental Figures**

**Figure-S1:**

***Tmem52 and lipolysis-related genes are activated under fat-burning conditions***

(**A**) Hierarchical clustered heatmap of gene expression profiles in iWAT of ctrl, run, swim and cold groups. (**B**) Venn diagram of overlapping DEGs. (**C**) Gene Ontology (GO) enrichment analysis based on the overlapping DEGs. (**D-E**) GSEA analysis based on the gene expression profiles in iWAT of ctrl, run, swim and cold groups.

**Figure-S2:**

***The body weight of Slc25a30, Gm4981 or 4930505A04Rik-KO mice is not remarkably changed***

(**A**) Schematic of the knockout strategy for *Tmem52* gene. (**B**) Body weight; WT, n = 8, leptolin-KO, n = 9. (**C**) Schematic of the knockout strategy for *Slc25a30* gene. (**D**) Body weight; WT, n = 8, *Slc25a30*-KO, n = 7. (**E**) Schematic of the knockout strategy for *Gm4981* gene. (**F**) Body weight; WT, n = 8, *Gm4981*-KO, n = 7. (**G**) Schematic of the knockout strategy for *4930505A04Rik* gene. (**H**) Body weight; WT, n = 8, *4930505A04Rik*-KO, n = 7. Data are mean ± s.e.m.; student's t-test was used for statistical analysis; *p < 0.05, n.s., not significant.

**Figure-S3:**

***Tmem52 has conserved amino acid sequence in mammals***

(**A**) Phylogenetic tree analysis showing *Tmem52* gene in mammal species. (**B**) Multiple alignment of leptolin of the mammal species.

**Figure-S4:**

***Tmem52 mRNA levels in adipose tissue are elevated under the condition of exercise and cold exposure***

(**A-C**) Relative mRNA expression of *Tmem52* in multiple organs and tissues in response to exercise and cold exposure; n = 4 per group. Data are mean ± s.e.m.; student's t-test was used for statistical analysis; *p < 0.05. (**D-F**) Single-cell level information of *TMEM52* in the Human Protein Atlas (HPA)-database (https://www.proteinatlas.org/ENSG00000178821-TMEM52/single+cell).

**Figure-S5:**

***Generation of Tmem52 RNAscope probe and anti-leptolin antibody***

(**A**) Schematic illustration shows the high sensitivity and specificity of RNAscope assay. (**B**) An immunogenic antigen is selected for the generation of a functional and highly specific anti-leptolin antibody.

**Figure-S6:**

***GEO datasets show that Tmem52 mRNA levels can be induced by exercise, cold stress and acute mechanical stress***

(**A-B**) Relative mRNA expression of *Tmem52* in adipose tissue of mice in the condition of running. (**C-D**) Relative mRNA expression of *Tmem52* in adipose tissue of mice in response to cold stress. (**E**) Relative mRNA expression of *Tmem52* in human cardiomyocytes in response to acute mechanical stress (cyclic mechanical stretch, 0.5 Hz, 10-21% elongation). (**F-G**) Relative mRNA expression of *Tmem52* in adipose tissue of mice in response to fasting. Data are mean ± s.e.m.; student's t-test was used for statistical analysis; *p < 0.05, n.s., not significant.

**Figure-S7:**

***Leptolin is an adipose tissue-derived protein induced by fat-burning conditions***

(**A**) Representative immunoblots of leptolin and tubulin from iWAT, eWAT and BAT, and the quantified ratio of leptolin/tubulin; n = 6 per group. (**B**) Representative immunofluorescence images of leptolin in iWAT; scale bar, 10 μm. (**C**) Representative immunofluorescence images of leptolin and VAMP2 in undifferentiated 3T3L1 cell; scale bar, 20 μm (left panel) and 5 μm (right panel); and percentage of intra-vesicular leptolin colocalized with VAMP2 in preadipocytes. (**D**) Representative immunofluorescence images of leptolin and VAMP2 in differentiated 3T3L1 cell; scale bar, 20 μm (left panel) and 1 μm (right panel); and percentage of intra-vesicular leptolin colocalized with VAMP2 in adipocytes. (**E**) SR-FACT technique detects the leptolin-EGFP signals in vesicles of live 293T cells transfected with plasmid. (**F**) Schematic illustration shows the roles of leptolin. Data are mean ± s.e.m.; the one-way ANOVAs were used for statistical analysis followed by Bonferroni’s post hoc test; *p < 0.05.

**Figure-S8:**

***Leptolin can be produced by adipocytes in vivo and in vitro***

(**A**) Representative RNAscope image visualizing *Tmem52* mRNA in adipocytes of iWAT; red dots were indicative of *Tmem52* mRNA signal; scale bar, 10 μm. (**B**) Representative immunofluorescence image of leptolin and VAMP2 in adipocytes of iWAT; scale bar, 10 μm. (**C-E**) Mature and immature 3T3L1 cells were treated with Cl316,243 for β3-adrenergic activation. (**C**) Relative mRNA expression (fold change) of *Tmem52* in 3T3L1 cell; n = 4 per group. (**D**) Representative immunoblots of leptolin and tubulin, and the quantified ratio of leptolin/tubulin; n = 4 per group. (**E**) Representative immunoblots of leptolin and images of Ponceau S staining from the medium of 3T3L1 cell; n = 4 per group. Data are mean ± s.e.m.; student's t-test was used for statistical analysis; *p < 0.05.

**Figure-S9:**

***Leptolin protein is not detected in serum of leptolin-KO mice, and is increased in serum of leptolin-TG mice***

(**A**) Representative immunoblots of leptolin and images of Ponceau S staining from serum of WT, leptolin-KO and leptolin-TG mice. (**B**) The quantified ratio of leptolin/Ponceau S; n = 7 per group. Data are mean ± s.e.m.; one-way ANOVA with Bonferroni test was used for statistical analysis; ***p < 0.001.

**Figure-S10:**

***Leptolin deficiency increases the susceptibility to HFD-induced obesity in mice at thermoneutrality***

(**A-D**) 8-week-old WT and leptolin-KO mice were housed at either 22°C or 30°C and fed a high-fat-diet. (**A**) Schematic illustration of experiments. (**B**) Body weight; WT 22°C, n = 8, Leptolin-KO 22°C, n = 9, WT 30°C, n = 9, Leptolin-KO 30°C, n = 9. (**C**) Fat mass (%). (**D**) Lean mass (%). n = 5 per group. Data are mean ± s.e.m.; one-way ANOVAs were used for statistical analysis followed by Bonferroni’s post hoc test; *p < 0.05, ***p < 0.001, n.s., not significant.

**Figure-S11:**

***Leptolin deficiency reduces EE***

(**A**) Indirect calorimetry was performed to quantify EE of WT and leptolin-KO mice during complete 24 hr light-dark cycles. (**B**) RER. (**C**) Motor activity. WT, n = 4, Leptolin-KO, n = 5. Data are mean ± s.e.m.; student's t-test was used for statistical analysis; *p < 0.05, **p < 0.01, n.s., not significant.

**Figure-S12:**

***Leptolin deficiency dampens glucose tolerance in mice***

(**A**) Schematic illustration of experiments. (**B**) Glucose tolerance test. (**C**) Insulin tolerance test. WT, n = 8, Leptolin-KO, n = 9. (**D**) Plasma insulin level; WT, n = 6, Leptolin-KO, n = 8. Data are mean ± s.e.m.; student's t-test was used for statistical analysis; *p < 0.05, **p < 0.01, ***p < 0.001.

**Figure-S13:**

***Leptolin deficiency elevates the triglyceride and total cholesterol levels in plasma***

(**A**) Schematic illustration of experiments. (**B**) Plasma triglyceride levels; n = 5 per group. (**C**) Plasma total cholesterol levels; WT, n = 6, Leptolin-KO, n = 7. Data are mean ± s.e.m.; student's t-test was used for statistical analysis; *p < 0.05.

**Figure-S14:**

***Adipocyte-specific Tmem52-knockout in iWAT dampens cold stress-induced weight loss***

(**A**) Schematic illustration of experiments. (**B**) Representative immunoblots of leptolin and tubulin, and the quantified ratio of leptolin/tubulin; n = 6 per group. (**C**) Representative immunoblots of leptolin and images of Ponceau S staining; n = 6 per group. (**D**) Fat pad weight; ctrl 22^o^C, n = 8, leptolin^Adi^ KO 22^o^C, n = 6; ctrl 4^o^C, n = 8, leptolin^Adi^ KO 4^o^C, n = 6. (**E**) Body weight; ctrl 22^o^C, n = 8, leptolin^Adi^ KO 22^o^C, n = 6; ctrl 4^o^C, n = 8, leptolin^Adi^ KO 4^o^C, n = 6. Data are mean ± s.e.m.; one-way ANOVAs followed by Bonferroni’s post hoc test were used for statistical analysis; *p < 0.05.

**Figure-S15:**

***Leptolin gene-overexpression increases EE***

(**A**) Indirect calorimetry was performed to quantify EE of WT and leptolin-TG mice during complete 24 hr light-dark cycles. (**B**) RER. (**C**) Motor activity. WT, n = 4, Leptolin-TG, n = 4. Data are mean ± s.e.m.; student's t-test was used for statistical analysis; *p < 0.05, n.s., not significant.

**Figure-S16:**

***Leptolin gene-overexpression improves glucose tolerance and insulin sensitivity in mice***

(**A**) Schematic illustration of experiments. (**B**) Glucose tolerance test. (**C**) Insulin tolerance test. WT, n = 6, Leptolin-TG, n = 6. (**D**) Plasma insulin level; WT, n = 5, Leptolin-TG, n = 5. Data are mean ± s.e.m.; student's t-test was used for statistical analysis; *p < 0.05, **p < 0.01, ***p < 0.001.

**Figure-S17:**

***Leptolin gene-overexpression reduces the plasma triglyceride levels***

(**A**) Schematic illustration of experiments. (**B**) Plasma triglyceride levels; WT, n = 5, Leptolin-TG, n = 5. (**C**) Plasma total cholesterol levels; WT, n = 5, Leptolin-TG, n = 8. Data are mean ± s.e.m.; student's t-test was used for statistical analysis; *p < 0.05.

**Figure-S18:**

***Screening workflow of human plasma sample and sWAT datasets***

(**A**) Schematic illustration of screening workflow of normal weight and obese humans, 55 individuals were included. (**B**) Schematic illustration of screening workflow of large sample human sWAT datasets with information of BMI, 2 datasets are included. (**C**) Scatter plots of mRNA levels of *TMEM52* and lipolysis-related genes in sWAT of differently normalized large sample human datasets; the red line shows correlation. (**D**) Schematic illustration of screening workflow of non-athletes and athletes, 32 individuals were included. (**E**) Scatter plots of the expression levels of *TMEM52* and genes associated with mitochondrial function and fat mobilization in sWAT of differently normalized Ctrl/Exercise human datasets; The red line shows correlation.

**Figure-S19:**

***The dose-effect study of leptolin I.P. injection in treating obesity***

(**A**) Schematic illustration of experiments: 8-week-old mice were fed a high-fat-diet, received PBS for 4 days as acclimation, then were intraperitoneally injected with PBS or mini-leptolin at dosages of 0.005-5 mg/kg/d daily for 2 months; PBS, n = 8, mini-leptolin 0.005 mg/kg/d, n = 9, mini-leptolin 0.05 mg/kg/d, n = 9, mini-leptolin 0.5 mg/kg/d, n = 8, mini-leptolin 5 mg/kg/d, n = 8. (**B**) Body weight. (**C**) Daily food intake. (**D**) Indirect calorimetry was performed to quantify EE during complete 24 hr light-dark cycles; n = 6 per group. Data are mean ± s.e.m.; one-way ANOVAs were used for statistical analysis followed by Tukey’s post hoc test; *p < 0.05, ***p < 0.001, n.s., not significant.

**Figure-S20:**

***Leptolin administration elevates energy expenditure and body surface temperature***

(**A**) Indirect calorimetry for quantifying EE of leptolin-treated mice during complete 24 hr light-dark cycles. (**B**) RER. n = 6 per group. (**C**) Motor activity. n = 5 per group. (**D**) Body surface temperature. n = 6 per group. Data are mean ± s.e.m.; student's t-test was used for statistical analysis; *p < 0.05, **p < 0.01, n.s., not significant.

**Figure-S21:**

***Leptolin administration induces gene expression associated with mitochondrial biogenesis and lipolysis in eWAT and BAT***

(**A**) Relative mRNA expression (fold change) of genes associated with mitochondrial biogenesis and lipolysis in eWAT; n = 4 per group. (**B**) Relative mRNA expression (fold change) of genes associated with mitochondrial biogenesis and lipolysis in BAT; n = 4 per group. Data are mean ± s.e.m.; student's t-test was used for statistical analysis; *p < 0.05.

**Figure-S22:**

***Leptolin administration improves glucose tolerance and insulin sensitivity in mice***

(**A**) Schematic illustration of experiments. (**B**) Glucose tolerance test. (**C**) Insulin tolerance test. n = 8 per group. (**D**) Plasma insulin level; PBS, n = 6, mini-leptolin, n = 8. Data are mean ± s.e.m.; student's t-test was used for statistical analysis; *p < 0.05, ***p < 0.001.

**Figure-S23:**

***Leptolin administration reduces triglyceride levels in plasma of HFD-induced obese mice***

(**A**) Schematic illustration of experiments. (**B**) Plasma triglyceride levels; n = 6 per group. (**C**) Plasma total cholesterol levels; PBS, n = 5, mini-leptolin, n = 7. Data are mean ± s.e.m.; student's t-test was used for statistical analysis; *p < 0.05, n.s., not significant.

**Figure-S24:**

***The cell size and lipid droplets of adipocytes are not markedly altered by leptolin treatment***

(**A**) Representative images of 3T3L1 cells; scale bar, 100 μm (left panel) and 50 μm (right panel); and cell diameters, n = 4 sections per group. (**B**) Representative immunofluorescence images of BODIPY in 3T3L1 cells; scale bar, 10 μm; and lipid droplets area, n = 4 sections per group. (**C**) Representative images of Oil Red O in 3T3L1 cells; scale bar, 50 μm (left panel) and 20 μm (right panel); and Oil Red O area, n = 4 sections per group. (**D**) Relative mRNA expression (fold change) of genes associated with mitochondrial biogenesis and lipolysis in 3T3L1 cells; n = 4 per group. (**E-F**) Relative mRNA expression (fold change) of *Adrb3* and *Dio2* in iWAT; n = 6 per group. (**G**) Representative images of IVIS SPECTRUM; and leptolin signal/area in multiple organs and tissues. (**H**) Illustration of leptolin actions. Data are mean ± s.e.m.; student's t-test was used for statistical analysis; *p < 0.05, n.s., not significant.

**Figure-S25:**

***The lipolysis-promoting effects of leptolin administration were dampened in sympathetically-denervated iWAT***

(**A**) Schematic illustration of workflow for experiments: the pharmacologic approach of 6-hydroxydopamine (6-OHDA) was employed to locally block the sympathetic signaling in iWAT of 8-week-old mice. Mice were fed a high-fat-diet, received PBS for 4 days as acclimation, then were intraperitoneally injected with mini-leptolin or PBS daily for 3 weeks, tissue harvest was performed at week 3. (**B**) Inguinal WAT weight; n = 6 per group. (**C**) Representative images of H&E staining of iWAT and the size profiling of adipocytes from iWAT; scale bar indicates 20 µm. (**D**) Relative mRNA expression of genes associated with mitochondrial function and fat mobilization in iWAT; n = 4 per group. (**E**) Schematic illustration of workflow for experiments: the pharmacologic approach of 6-OHDA was employed to locally block the sympathetic signaling in iWAT of 8-week-old mice. Mice were fed a high-fat-diet, received PBS for 4 days as acclimation, then were intracerebroventricularly injected with mini-leptolin or PBS daily for 3 weeks, tissue harvest was performed at week 3. (**F**) Inguinal WAT weight; n = 6 per group. (**G**) Representative images of H&E staining of iWAT and the size profiling of adipocytes from iWAT; scale bar indicates 20 µm. (**H**) Relative mRNA expression of genes associated with mitochondrial function and fat mobilization in iWAT; n = 4 per group. Data are mean ± s.e.m.; one-way ANOVAs followed by Bonferroni’s post hoc test were used for statistical analysis; *p < 0.05.

**Figure-S26:**

***Original full western blot images***

**Table-S1.** **Primers used in the present study**

| **Primer** | **Forward Primer 5'-3'** | **Reverse Primer 5'-3'** |
| --- | --- | --- |
| *Tmem52* | TGCTCGCCATACTCCTGATG | CTGTGTGCGGGGCTATCAC |
| *Ppargc1a* | AGCCGTGACCACTGACAACGAG | GCTGCATGGTTCTGAGTGCTAAG |
| *Cox8b* | GAACCATGAAGCCAACGACT | GCGAAGTTCACAGTGGTTCC |
| *Cox7a1* | CAGCGTCATGGTCAGTCTGT | AGAAAACCGTGTGGCAGAGA |
| *Ucp1* | ACTGCCACACCTCCAGTCATT | CTTTGCCTCACTCAGGATTGG |
| *Prdm16* | CAGCACGGTGAAGCCATTC | GCGTGCATCCGCTTGTG |
| *Plin1* | GGGACCTGTGAGTGCTTCC | GTATTGAAGAGCCGGGATCTTTT |
| *Slc27a1* | CGCTTTCTGCGTATCGTCTG | GATGCACGGGATCGTGTCT |
| *Slc27a2* | TCCTCCAAGATGTGCGGTACT | TAGGTGAGCGTCTCGTCTCG |
| *Lipe* | GATTTACGCACGATGACACAGT | ACCTGCAAAGACATTAGACAGC |
| *Pnpla2* | GGATGGCGGCATTTCAGACA | CAAAGGGTTGGGTTGGTTCAG |
| *Acsl1* | TGCCAGAGCTGATTGACATTC | GGCATACCAGAAGGTGGTGAG |
| *Srebf1* | TGACCCGGCTATTCCGTGA | CTGGGCTGAGCAATACAGTTC |

**Video-S1.** **SR-FACT technique detects the leptolin-EGFP signals in vesicles of live human 293T cells** **transfected with plasmid, related to FigureS5**
